# Can milk cell or skim milk miRNAs be used as biomarkers for early pregnancy detection in cattle?

**DOI:** 10.1371/journal.pone.0172220

**Published:** 2017-02-24

**Authors:** Corina I. Schanzenbach, Benedikt Kirchner, Susanne E. Ulbrich, Michael W. Pfaffl

**Affiliations:** 1 Animal Physiology and Immunology, Department of Animal Sciences, Wissenschaftszentrum Weihenstephan, Technische Universität München, Freising, Germany; 2 Animal Physiology, Institute of Agricultural Science, Department of Environmental Systems Science, ETH Zürich, Zurich, Switzerland; Huazhong University of Science and Technology, CHINA

## Abstract

The most critical phase of pregnancy is the first three weeks following insemination. During this period about 50% of high yielding lactating cows suffer embryonic loss prior to implantation, which poses a high economic burden on dairy farmers. Early diagnosis of pregnancy in cattle is therefore essential for monitoring breeding outcome and efficient production intervals. Regulated microRNAs (miRNAs) that reach easily accessible body fluids via a ‘liquid biopsy’ could be a new class of pregnancy predicting biomarkers. As milk is obtained regularly twice daily and non-invasively from the animal, it represents an ideal sample material. Our aim was to establish a pregnancy test system based on the discovery of small RNA biomarkers derived from the bovine milk cellular fraction and skim milk of cows. Milk samples were taken on days 4, 12 and 18 of cyclic cows and after artificial insemination, respectively, of the same animals (n = 6). miRNAs were analysed using small RNA sequencing (small RNA Seq). The miRNA profiles of milk cells and skim milk displayed similar profiles despite the presence of immune cell related miRNAs in milk cells. Trends in regulation of miRNAs between the oestrous cycle and pregnancy were found in miR-cluster 25~106b and its paralog cluster 17~92, miR-125 family, miR-200 family, miR-29 family, miR-15a, miR-21, miR-26b, miR-100, miR-140, 193a-5p, miR-221, miR-223, miR-320a, miR-652, miR-2898 and let-7i. A separation of cyclic and pregnant animals was achieved in a principal component analysis. Bta-miRs-29b, -221, -125b and -200b were successfully technically validated using quantitative real-time PCR, however biological validation failed. Therefore we cannot recommend the diagnostic use of these miRNAs in milk as biomarkers for detection of bovine pregnancy for now.

## Introduction

The time of pregnancy establishment is the most critical phase during reproduction as about 50% of lactating cattle suffer embryonic loss during the first three weeks of gestation [1]. For the dairy industry, this results in significant financial losses. Thus, in modern dairy farms, early diagnosis of pregnancy in cattle is essential for monitoring breeding outcomes as early as possible. To date a reliable detection of pregnancy in dairy cows remains topical as all existing pregnancy testing methods have limits in either sensitivity, accuracy, practicality or timing [2]. Some methods, such as ultrasonography or pregnancy associated glycoproteins *(PAG)* test are very reliable for pregnancy detection or in case of the milk progesterone for detection of non-pregnancy [2]. However unfortunately these tests are not applicable before four or three weeks, respectively, post insemination. In case of non-pregnancy this is too late for repeated breeding, since a recurrent oestrus cycle has already started. A novel biomarker indicating pregnancy before day 20 after insemination would be of great advantage. Preferably it would enable a quick diagnosis independent of the cattle breed, the feeding or the environment and would be easily accessible, ideally non-invasively taken.

In recent years the novel class of non-coding RNAs, namely micro RNAs (miRNAs), has been extensively studied. These non-coding RNAs are 16–27 nt long and play an outstanding role in post-transcriptional gene expression regulation [3]. Mature miRNAs can bind complementarily onto the 3’-untranslated region of a target mRNA and thus either inhibit its translation or lead to destabilization and degradation of the bound mRNA [3, 4]. miRNAs are found in practically all body fluids, including blood [5], saliva [6], urine [7] and milk [8]. Their unique expression pattern in various diseases and the fact that they can be sampled in a non- or minimally invasive manner, also known as liquid biopsies, make them ideal molecules for diagnostic biomarkers [9–11].

As pregnancy is governed by distinct regulatory processes, it is unsurprising to find miRNAs are highly expressed in the uterus and associated reproduction active organs [12–14]. Hormones, such as progesterone and estrogen, are known to control the generally-repressed miRNA expression by interacting with nuclear steroid receptors which have the ability to influence mRNA and miRNA processing and bioavailability [15–17]. To date, miRNA involvement has been found in a broad range of reproductive processes including the immunological recognition of the embryo, implantation and oocyte maturation [18–20]. Regulated miRNAs that reach easily accessible body fluids therefore might be a new class of pregnancy predicting biomarkers.

Milk is a body fluid that is very easily accessible as it is routinely sampled several times daily in dairy cattle. As miRNAs are known to be highly abundant in milk, this would be an ideal biological sample for routine pregnancy diagnosis testing [21]. The entry of miRNAs into the milk may take place in predominantly two ways. Blood-derived miRNAs may either reach the milk via diapedesis of miRNA expressing immune cells [22] or via extracellular vesicles, e.g. exosomes, released into the milk by the alveolar mammary epithelia cells [23]. Exosomes are very stable membrane-covered vesicles (40–150 nm) released by a large variety of cells into extracellular compartments. They are packaged with proteins, mitochondrial DNA, mRNA and a high density of miRNAs. Hence, they represent an ideal transport shuttle for information throughout the body. Today the exosome crosstalk is thought to resemble a para- or endocrine like communication and microvesicles are increasingly used in biomarker development [24, 25].

As both immune cells and exosomes may carry information derived from the blood system, our hypothesis is that the miRNA profile is altered by the fundamental changes that occur in the maternal system during the early phase of pregnancy.

The goal of this study was to investigate different time points during oestrus cycle and after insemination: 1^st^) day 4, the time when the 16-cell stage embryo is entering the uterus; 2^nd^) day 12, when the embryo starts elongation and 3^rd^) day 18, when pregnancy recognition takes place. At this time the expression of the anti-luteolytic pregnancy recognition signal Interferon tau is at a peak and the maternal system protects the semi-allogenic conceptus from an inflammatory response by a local reaction that is protecting the embryo [26, 27].

The potential of miRNA biomarkers for early pregnancy detection serving as a ‘biomarker signature’ in milk was analysed in a holistic small RNA transcriptome screening of the milk cellular fraction (MC) and the skim milk fraction (SM), using the hypothesis free method of small RNA sequencing (small RNA Seq).

## Materials and methods

### Milk sample collection

Milk samples were taken from randomly chosen, healthy, lactating Brown Swiss cows (n = 10) housed at the research station Veitshof (located at Technical University Munich, Weihenstephan) on days 4, 12 and 18 of oestrous cycle and after artificial insemination of a subsequent oestrous cycle, respectively. Only cows that were routinely artificially inseminated during their lactation period at the research station were included into the study. The animals were housed in a freestall barn and care of the animals was performed in accordance with good livestock farming practices by the research station facility staff. All cows had water access ad libitum and were fed a daily basic ratio comprising 22 kg corn silage, 10 kg grass silage and 2 kg hay. 2 kg high-protein crushing rape and soya (deuka Kompopur 404, Deutsche Tiernahrung Cremer, Germany) and 125 g mineral mix (Josera, Germany) were supplemented to the diet in order to maintain energy equilibration and mineral balance, respectively. 0.5 kg concentrate (deuka, MK 194-UDP; Deutsche Tiernahrung Cremer) per delivered liter milk were added in order to meet performance related requirements of the dairy cows. The progesterone concentrations in the animals’ milk was monitored [28] and oestrous behaviour was observed to define oestrous as day 0. Pregnancy was achieved by artificial insemination with seminal plasma at the day of standing oestrous. Milk samples were collected during routine milking as total quarter milk and stored at 4°C after collection until further processing within the next three hours. 450ml of fresh milk was centrifuged at 1500 x g at 4°C for 30 min. The fat layer was discarded and the SM collected and frozen at -20°C until further processing. The obtained cell pellet, containing all MC including the milk somatic cells and exfoliated mammary gland epithelial cells, was washed in 50 ml cold phosphate buffered saline (137 mM sodium chloride, 2.7 mM potassium chloride, 7.3 mM monopotassium phosphate, 8.1 mM disodium phosphate dihydrate, pH 7.4) followed by centrifugation at 1500 x g and 4°C for 15 min. Afterwards the cell pellet was stabilized in 1 ml Qiazol lysis reagent (Qiagen, Germany) in a shredder tube. The cells were homogenized for 30 sec at 7000 rpm in a MagNa Lyser (Roche Instrument Center, Switzerland) and frozen at -80°C until further processing.

### Total RNA isolation

Total RNA of MC and SM, including mRNA and miRNA, was isolated using a phenol chloroform based method. MC total RNA was isolated column-free by adding 100 μl chloroform to 1 ml Qiazol (Qiagen, Germany), incubating, vortexing and centrifuging (15 min, 13,400 x g, 4°C). The aqueous phase was then mixed with the same volume of ice cold isopropanol and washed twice with 75% EtOH (5 min, 13,400 x g, 4°C). Supernatant was removed and remaining EtOH evaporated. The pellet was redissolved in RNAse free H_2_O, heated at 68°C for 2 min and stored at -80°C until further processing. The lower concentrated SM total RNA was isolated on-column with the miRNeasy Mini Kit (Qiagen, Germany). The higher fat content in milk required higher Qiazol lysis reagent amounts than the original volume suggested by the manufacturer. Thus, 12 ml Qiazol was used to extract RNA from 6 ml SM. Aside from the afore-mentioned exception, the procedure was followed according to manufacturer’s protocol (Qiagen, Germany). Isolated pure total RNA was eluted with RNAse free H_2_O. RNA integrity and miRNA content of all RNA eluates were assessed using a small RNA Chip and a RNA 6000 Nano Chip on a Bioanalyzer 2100 (Agilent Technologies, CA, USA). The RNA samples were stored at -80°C until further processing.

### Small RNA sequencing

In preparation for next generation sequencing (NGS) a total of 72 RNA samples from n = 6 animals for MC and SM samples, respectively were prepared in a library preparation using NEBNext Multiplex Small RNA Library Prep Set for Illumina (New England BioLabs, MA, USA) described by our group previously [29]. In brief, adaptor sequences were ligated to 200 ng total RNA starting material. The RNA was reverse transcribed, amplified by PCR and barcoded for 24x multiplexing. The DNA concentrations of all samples were determined using a DNA 1000 chip on a Bioanalyzer 2100 (Agilent Technologies, CA, USA). The samples were pooled and fractions were size-selected via high resolution gel electrophoresis. The gel bands were cut at 135–151 bp, corresponding to small RNA size of 16–32 bp. The DNA libraries were controlled regarding correct size, purity and concentration with a High Sensitivity DNA chip on a Bioanalyzer 2100. Single-read sequencing-by-synthesis was carried out in 50 cycles on a HiSeq 2500 platform (Illumina, CA, USA) using the TruSeq^®^ Rapid SBS Kit—HS (50 cycle) and TruSeq^®^ Rapid SR Cluster Kit—HS (Illumina). Each flow cell was loaded with each 12 MC and 12 SM samples to assure reproducible conditions.

### Data processing, mapping and annotation

The resulting data was processed using an in-house procedure [29]. Adaptor sequences were trimmed from 3’end with Btrim [30]. The sequence data quality control software FastQC (Babraham Bioinformatics, UK, Version 0.10.1) was used to calculate length distribution and representing base calling accuracy by the phred quality scores (Q score). For prevention of bias and false-positive mappings by degenerated RNA or other material all reads shorter than 16 nt were depleted. Remaining reads were mapped to RNAcentral database [31] to be filtered for bovine rRNA, tRNA, snRNA and snoRNA sequences. For filtering, one mismatch in the first 15 nt and a variable number of mismatches in the remaining sequence were allowed. The resulting dataset was aligned to the most recent miRBase database (release 21) [32] for mature bovine miRNAs using Bowtie short read aligner [33]. Default parameters were used except of the “best” alignment algorithm, which was adapted by only allowing one mismatch in the whole sequence. Due to RNA properties no alignment was performed on the reverse complementary strand. All aligned reads were sorted and indexed by SAMtools [34] and readcounts finally generated by calling the sum of hits per miRNA sequence. Readcounts were separately normalized for MC and SM samples using the DESeq2 R script (R version 3.1.2)[35]). A noise-cut-off was performed for miRNAs with average readcounts <50. Statistical analysis was performed using DeSeq2 for readcount and paired t-test for fold change comparison as well as Pearson correlation in SigmaPlot (Systat Software, IL, USA, Version 12.0.0.182). P-values < 0.05 were considered as significant, p-values < 0.01 as highly significant. Principal component analysis (PCA) was carried out using GenEx software (GenEx Pro, version 5.4., Multid Analyses AB, Sweden). The Venn Diagram was generated using the web application BioVenn by Tim Hulsen [36].

### Reverse Transcription and qPCR measurement (RT-qPCR)

The RT-qPCR validation of NGS results was performed using the miScript PCR System (Qiagen). For quantification of miRNAs in MC 300 ng of total RNA (n = 10 animals) were reversed transcribed using HighSpec buffer. Six of the ten animals analysed were included in the sequencing study previously and thus served as technical validation, four additional animals from the same trial were only analyzed in RT-qPCR and are therefore considered as biological validation. The qPCR was carried out in the CFX 384 Real-Time PCR Detection System (Bio-Rad, CA, USA). The following miScript assays were used: Bt_mir-25_1, Bt_mir-106b_1, Mm_mir-93_1, Hs_mir-221_1, Bt_mir-186_1, Bt_let-7i_1, Bt_mir-125b_1, Bt_mir-200b_1, Bt_mir-200c_1, Bt_mir-193a-5p_1 and Bt_mir-2898_1. For SM reverse transcription 200 ng of total RNA of n = 6 animals (the samples of all six animals were included in the sequencing study and therefore serve as technical validation) was carried out and quantified as mentioned above using the miScript assays: Bt_mir-92a_1, Hs_mir-20a_1 and Mm_mir-29b_1. For MC and SM samples, respectively, negative controls lacking either RNA material or RT enzyme were used during RT, a RNA pool of all MC and SM samples, respectively, served as a positive control. These controls and an additional water control, lacking cDNA, were included in all RT-qPCR measurements. In order to identify the best reference miRNAs for assay normalization, three stable expressed miRNAs without regulation and reads >1500 were selected from the NGS data set to be analysed using GenEx software (GenEx Pro, MultiD, Gothenburg, Sweden) using the Normfinder Tool [37]. For MC the miScript assays Hs_mir-27b_1, Bt_mir-181a_1 and Hs_RNU6-2_1, and for SM Mm_mir_26a_1, Bt_mir-186_1 and Bt_mir-148a_1 were used for normalization. Relative gene expression data was analysed using the method described by Pfaffl and following the MIQE guidelines [38, 39].

## Results

### Small RNA sequencing

Total RNA was extracted from MC and SM to construct a library for next generation sequencing with Illumina HiSeq 2500. The RNA integrity number (RIN) of MC samples was 6.1 ± 0.6, the RIN of SM was 2.3 ± 0.6. Reverse-transcribed and barcoded small RNA was size selected for 135 bp to 151 bp. The Illumina run resulted in 16.8 ± 5.8 m of high quality reads (> 98% of all reads have a phred score > 30) for MC and 14.8 ± 2.2 m for SM of which 7.0 ± 3.1 m and 5.0 ± 1.5 m, respectively, were longer than 15 nt. 3.7 ± 1.9 m reads in MC and 3.0 ± 0.9 m in SM were mapped to rRNA, snRNA, snoRNA and tRNA using RNAcentral database [31] and consequently filtered from the dataset. From the remaining reads 318 ± 183 k and 149 ± 70 k reads were mapped to miRBase and were thus used for miRNA analysis.

### miRNAs expressed in milk cells and skim milk

**Fig 1:**
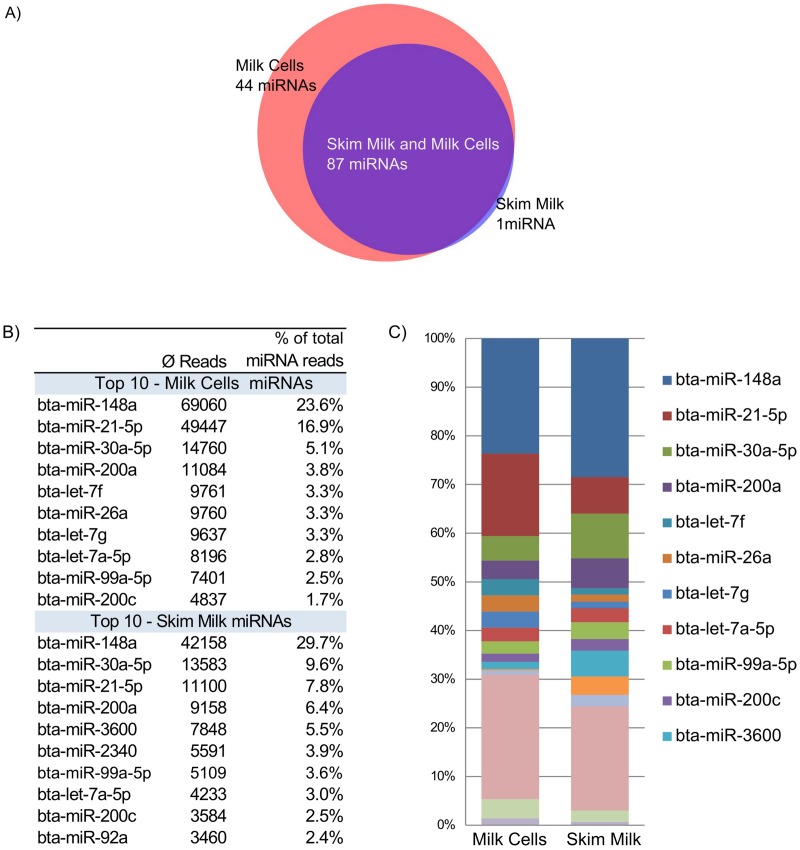
miRNA distribution in Milk Cells (MC) and Skim Milk (SM). A) Venn diagram of miRNAs >50 reads overlapping or discriminating between MC and SM, respectively. B) Top ten highest expressed miRNAs both in MC and SM. DESeq2 normalized reads and corresponding ratio to total miRNA reads are shown. C) Graphical illustration of miRNA distribution among MC and SM samples.

In total 335 miRNAs were found both in MC and SM samples with more than one read on average. For removal of imprecise background reads only miRNAs with more than 50 reads in average were considered in the continuing analysis. This resulted in 132 different miRNAs with more than 50 reads. Even though about twice as many raw reads mapped to miRNAs in MC compared to SM, the majority of miRNAs (87 miRNAs) were found in both milk fractions. Additionally, 44 miRNAs were exclusively found in MC, representing around one third of all miRNAs, whilst only one miRNA was found in SM but not in MC (Fig 1A, S1 Table).

The analysis of the ten most highly expressed miRNAs showed a similar pattern. Seven of the top ten miRNAs match MC and SM (bta-miR 148a, bta-miR-21-5p, bta-miR-30a-5p, bta-miR-200a, bta-miR-99a-5p, bta-let-7a-5p, bta-miR-200c, Fig 1B and 1C), respectively. Most of the miRNAs with highest expression (except of bta-mir-3600 and bta-mir-2340) were described as unique or having higher expression levels in milk compared to serum samples by Chen et al. 2010 and were among the top 30 expressed miRNAs of milk glandular cells as described in a study by Guillo et al. 2014 [21, 40]. The top ten miRNAs made up 66.3% and 74.4% of all normalized miRNA reads in MC and SM, respectively.

#### Expression level correlation between milk cell miRNAs and skim milk miRNAs

**Fig 2:**
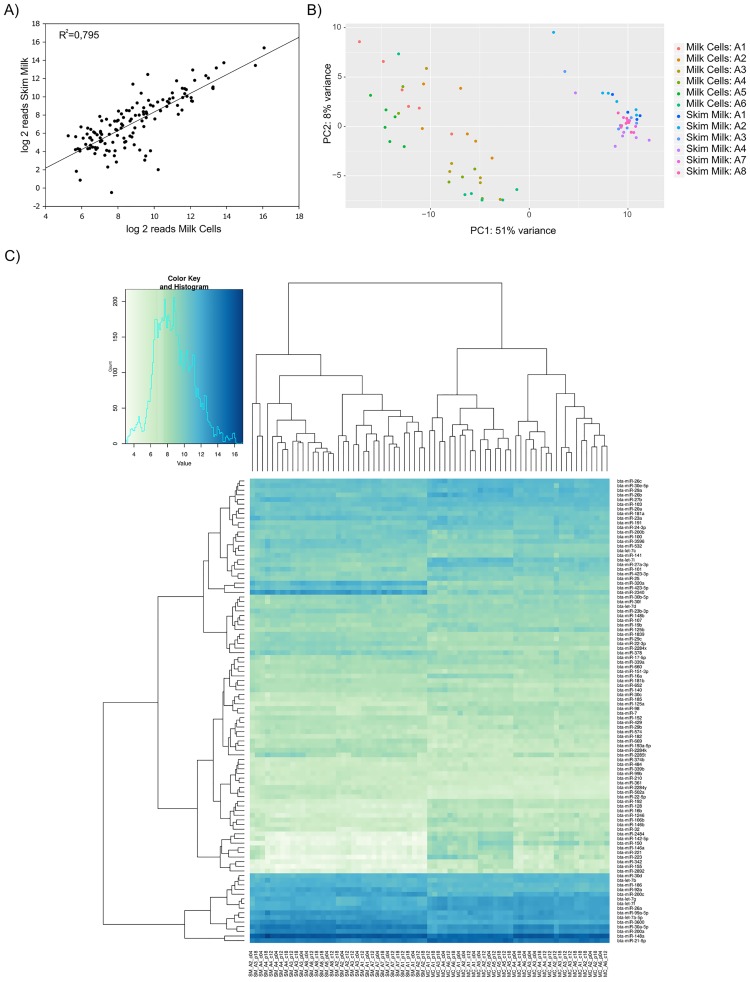
Comparison of MC and SM miRNA profile. A) Correlation between miRNA mean values of all MC and SM NGS reads. Normalized NGS reads were used for calculation of Pearson Correlation Coefficient B) Heatmap of the top 100 miRNAs. Data was normalized, rld transformed and clustered using DESeq2 C) PCA showing MC and SM specific clustering. Clustering was performed using DESeq2.

The average miRNA expression levels correlated between MC and SM with a highly significant (p = 4.8 E-30) correlation coefficient of 0.795 (Fig 2A). Still, the two samples types separated in PCA and hierarchical clustering as can be seen in Fig 2B and 2C. Additionally, the sample clustering shows that the SM samples were much more homogenous than MC samples. Those miRNAs responsible for the separation of the two milk fractions in the PCA were highly expressed miRNAs, such as miR-148a or miR-21-5p, however also less abundant but instead highly differently expressed miRNAs between MC and SM (Table 1). Most of these miRNAs are described as blood cell specific [41, 42]. However, these blood cell specific miRNAs make up less than 5% of the total readcounts in MC while about 75% belonged to the miRNAs expressed by the mammary gland [21, 40].

**Table 1:** Statistically significant differences in miRNA levels between milk cells and skim milk.

miRNA	Likely origin	baseMean	baseMean
		Milk Cells	Skim Milk
**bta-miR-150**	**Immune cells**	**803**	**6**
bta-miR-451	Erythrocytes	135	1
bta-miR-2484		481	12
bta-miR-142-5p	Immune cells	514	17
bta-miR-223	Im. cells/Erythr.	618	24
bta-miR-222	Immune cells	120	7
bta-miR-146a	Immune cells	406	26
bta-miR-155	Immune cells	164	11
bta-miR-342		151	17
bta-miR-221	Immune cells	330	39
bta-miR-128		223	32
bta-miR-15b	Im. cells/Erythr.	106	18
bta-miR-2892		151	26
bta-miR-2332		123	24
bta-miR-320a		295	4033
bta-miR-2340		531	8511

All significant different expressed miRNAs with expression ratios >5 between milk cells and skim milk are shown (n = 23), likely origin according to Undi 2013 and Sonkoly 2008 [41, 42]

Even though the overall miRNA expression level showed good correlation between SM and MC, the picture is more complex when looking at individual miRNAs from corresponding samples of the identical whole milk sample. While some miRNAs significantly correlated between both milk fractions, others did not (Table 2). Immune cell related miRNAs, such as miR-146a, miR-155, miR-221 or miR-15b, as well as highly expressed milk specific miRNAs like miR-148a, miR-221-5p, miR-30a-5p, miR-200a, miR-99a-5p, miR-7f demonstrated a significant correlation while approximately half of the highest expressed miRNAs (let-7a-5p, miR-26a, miR-200c, let-7f, miR-92a) were not significantly correlated between both milk fractions.

**Table 2:** Correlation of miRNA expression between milk cells and skim milk.

miRNA	Correlation Coefficient	Pearson correlation P-value	Likely origin	Regulated in pregnancy
miR-148a	**0.574**	**0.004**	Mammary Gland	TOP 20 MC+SM
miR-21-5p	**0.483**	**0.020**	Mammary Gland	TOP 20 MC+SM
miR-30a-5p	**0.473**	**0.023**	Mammary Gland	TOP 20 MC+SM
miR-200a	**0.593**	**0.003**	Mammary Gland	TOP 20 MC+SM
miR-99a-5p	**0.600**	**0.003**	Mammary Gland	TOP 20 MC+SM
miR-3600	**0.487**	**0.018**		TOP 20 MC+SM
let-7a-5p	-0.238	0.273	Mammary Gland	TOP 20 MC+SM
miR-26a	-0.224	0.305	Mammary Gland	TOP 10 MC+SM
miR-200c	-0.218	0.318	Mammary Gland	TOP 10 MC+SM; P
let-7f	-0.017	0.939	Mammary Gland	TOP 10 MC+SM
let-7g	**0.517**	**0.012**	Mammary Gland	TOP 10 MC+SM
miR-92a	0.043	0.846	Mammary Gland	TOP 10 SM; P
miR-2340	-0.257	0.236		TOP 10 SM; MC/SM
miR-2484	-0.031	0.889		MC/SM
miR-320a	**0.478**	**0.021**		MC/SM; P
miR-142-5p	0.328	0.126	Immune cells	MC/SM
miR-222	0.373	0.079	Immune cells	MC/SM
miR-150	0.377	0.076	Immune cells	MC/SM
miR-146a	**0.785**	**9.30E-06**	Immune cells	MC/SM
miR-155	**0.687**	**3.00E-04**	Immune cells	MC/SM
miR-221	**0.571**	**0.004**	Immune cells	MC/SM; P
miR-15b	**0.556**	**0.006**	Im. cells/Erythr.	MC/SM
miR-223	0.409	0.053	Im. cells/Erythr.	MC/SM
miR-451	-0.064	0.774	Erythrozytes	MC/SM
miR-342	0.257	0.236		MC/SM
miR-128	-0.011	0.959		MC/SM
miR-2892	0.112	0.612		MC/SM
miR-2332	0.341	0.111		MC/SM
let-7i	0.322	0.134		P
miR-20a	0.253	0.245		P
miR-200b	-0.123	0.576	Mammary Gland	P
miR-29b	**0.562**	**0.005**		P
miR-93	0.343	0.110		P
miR-193a-5p	**0.507**	**0.014**		P
miR-2898	0.165	0.452		P
miR-125b	0.263	0.225		P
miR-25	0.106	0.629		P
miR-106b	0.332	0.122		P
miR-140	0.379	0.074		P
miR-100	0.225	0.302		P
miR-125a	0.129	0.556		P
miR-223	0.409	0.052		P
miR-26b	**0.503**	**0.015**		P
miR-29c	**0.417**	**0.048**		P

Correlation coefficient and p-value between milk cells and skim milk of miRNAs which are either among the top 20 highest expressed (Top 20), differentially expressed between milk cells and skim milk (MC/SM) or differentially expressed during pregnancy (P) are shown. Significant correlations are indicated in bold. Likely origin according to Undi 2013 and Sonkoly 2008 [41, 42], n = 23.

### High throughput sequencing: miRNA regulation during early pregnancy

Overall there were few pregnancy-related expression changes, however clear trends in regulation were present in several miRNAs. Only miRNAs with more than 100 reads were analyzed in regard to the use as biomarkers because a routine measurement with RT-qPCR would require a minimum input of starting material for stable measurement. The miRNAs defined as regulated had to show an average fold-change higher than 1.4 or lower than -1.4, respectively, between pregnant and cyclic states and a p-value smaller than 0.05. It must be noted that no correction for multiple testing was performed on the p-values, although we are aware that this increases the risk of false positive significances. Such a correction would have led to a higher stringency and would have masked many of the differences that we found during the oestrus cycle and pregnancy progress.

**Fig 3:**
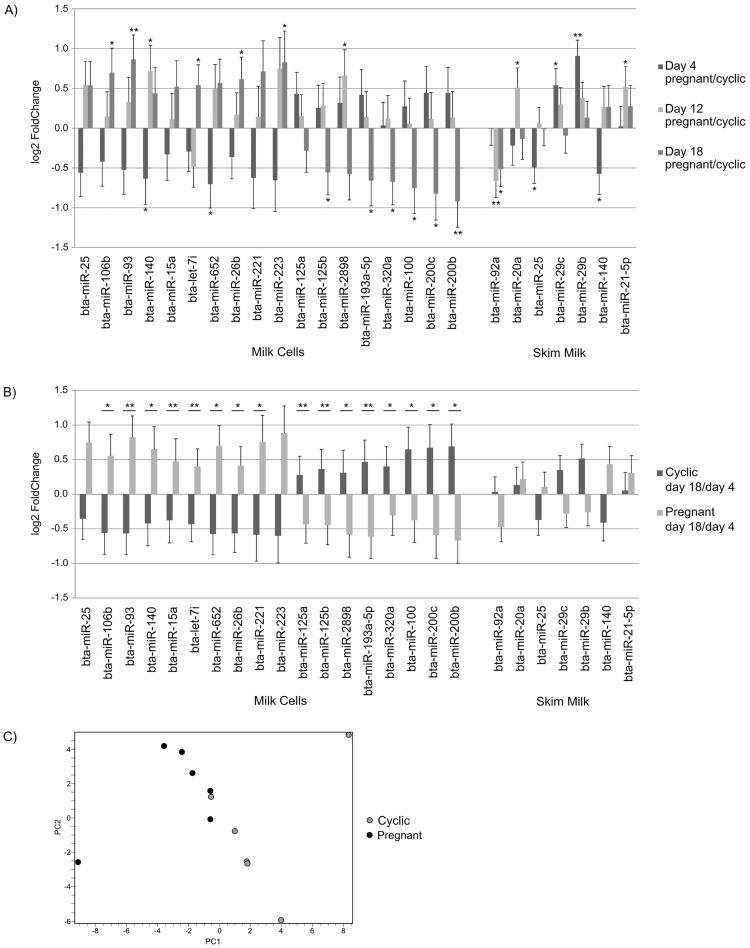
log2 fold-change of selected miRNAs in the milk cellular and skim milk fraction measured with high throughput sequencing. log2 fold-change of selected miRNAs in the milk cellular and skim milk fraction (n = 6, except SM pregnant day 18 n = 5) measured with high throughput sequencing. All fold-changes and p-values for pregnant/cyclic data were calculated using Deseq2. p-values between cyclic (18c/4c) and pregnant Day 18/Day 4 ratios (18p/4p) were calculated using paired t-test on log2 fold-changes *: p<0.05, **: p< 0.01. Error bars are shown as log fold standard error. A) log2 fold-change between pregnancy and oestrous cycle on days 4, 12 and 18 B) log2 fold-change between Day 18 and Day 4 during oestrous cycle and pregnancy. C) PCA of log2 fold-change of the miRNAs: bta-miR-25, bta-miR-93, bta-miR-106b, bta-miR-125b, bta-miR-193a-5p, bta-miR-200b, bta-miR-200c, bta-miR-221, bta-miR-2898, bta-let-7i between days 4 and 18 (Day 18/Day 4) showing the separation of cyclic and pregnant animals.

In the MC fraction 18 miRNAs (up regulation: Bta-miR-25, -106b, -93, -140, 15a, -652, -26b, -221, -223, bta-let-7i, down regulation: Bta-miR-125a, -125b, -2898, -193a-5p, 320a, 100, -200c, -200b) and in SM 7 miRNAs (Bta-miR-92a, -20a, -25, -29b, -29c, -140, 21-5p) were found to be regulated (Fig 3A and 3B, Table 3). Note that only miRNAs bta-miR-25 and bta-miR-140 were found to be regulated in SM as well as in MC.

**Table 3:** Fold-change/ratio of regulated miRNA during oestrous cycle (cy) and pregnancy (p) measured in NGS.

miRNA			Fold-change (NGS)				
	Reads		Day			Day	
	MC	SM	4p/4cy	12p/12cy	18p/18cy	18cy/4cy	18p/4p
**Regulation in Milk Cells**							
bta-miR-25	1302	360	-1.48	1.46	1.45	-1.27	1.68
bta-miR-106b	406	52	-1.34	1.11	**1.62**	-1.48 **(a)**	1.47 **(a)**
bta-miR-93	138	36	-1.44	1.25	***1*.*82***	-1.47 ***(a)***	*1*.*76* ***(a)***
bta-miR-140	406	163	**-1.55**	**1.64**	1.35	-1.34 **(a)**	1.57 **(a)**
bta-miR-15a	106	14	-1.26	1.08	1.44	-1.23 ***(a)***	1.39 ***(a)***
bta-let-7i	2975	374	-1.22	-1.40	**1.45**	-1.35 ***(a)***	1.31 ***(a)***
bta-miR-652	225	161	**-1.63**	1.41	1.48	-1.49 **(a)**	1.61 **(a)**
bta-miR-26b	4434	731	-1.28	1.12	**1.53**	-1.48 **(a)**	1.32 **(a)**
bta-miR-221	476	27	-1.54	1.10	1.64	-1.50 **(a)**	1.68 **(a)**
bta-miR-223	890	17	-1.58	1.68	**1.78**	-1.52	1.84
bta-miR-125a	437	48	1.35	1.11	-1.22	1.21 ***(a)***	-1.35 ***(a)***
bta-miR-125b	1052	190	1.19	1.21	**-1.47**	1.29 ***(a)***	-1.36 ***(a)***
bta-miR-2898	131	34	1.24	**1.58**	-1.49	1.24 **(a)**	-1.50 **(a)**
bta-miR-193a-5p	137	166	1.34	1.10	**-1.58**	1.38 ***(a)***	-1.53 ***(a)***
bta-miR-320a	453	2680	1.02	1.09	**-1.60**	1.32 **(a)**	-1.24 **(a)**
bta-miR-100	1170	686	1.21	1.04	**-1.69**	1.57 **(a)**	-1.30 **(a)**
bta-miR-200c	4837	3584	1.36	1.08	**-1.77**	1.59 **(a)**	-1.51 **(a)**
bta-miR-200b	1697	719	1.36	1.10	***-1*.*89***	1.61 **(a)**	-1.59 **(a)**
**Regulation in Skim Milk**							
bta-miR-92a	2419	3460	-1.00	***-1*.*58***	**-1.43**	1.02	1.39
bta-miR-20a	2101	947	**-1.16**	**1.42**	-1.10	1.10	-1.16
bta-miR-25	1302	360	**-1.41**	1.04	-1.01	1.30	-1.08
bta-miR-29c	394	356	**1.45**	1.23	-1.07	1.27	1.22
bta-miR-29b	307	116	***1*.*88***	1.30	1.10	-1.43	1.20
bta-miR-140	406	163	**-1.49**	1.20	1.20	1.33	-1.35
bta-miR-21-5p	49447	11100	1.01	**1.43**	1.21	-1.04	-1.24

Fold-changes/ratios of miRNAs in the milk cellular and skim fraction (n = 6, except SM pregnant Day 18 n = 5) measured with high throughput sequencing. All fold-changes (p/c and Day 18/Day 4) and p-values for p/c data were calculated using Deseq2. p-values between cyclic and pregnant Day 18/Day 4 ratios were calculated using paired t-test on log2 fold-changes. Significant values (p<0.05) are indicated in bold, highly significant values (p<0.01) in bold italic. Significant differences between cyclic and pregnant Day 18/Day 4 fold-changes are indicated with letter (a) in bold or bold italic.

Two major findings were observed in MC sequencing data. On the one hand the expression ratio of all miRNAs between cyclic and pregnant states increased or decreased continously over time (Fig 3A, Table 3), respectively. On the other hand the ratio Day 18/Day 4, the timepoints furthest apart, thus showing the biggest differences, was always complementary between the cyclic and pregnant state (Fig 3B, Table 3). Consequently, Day 18/Day 4 ratios of cyclic (18c/4c) and pregnant animals (18p/4p) of a set of miRNAs (bta-miR-25, bta-miR-93, bta-miR-106b, bta-miR-125b, bta-miR-193a-5p, bta-miR-200b, bta-miR-200c, bta-miR-221, bta-miR-2898, bta-let-7i) were used in a PCA. This analysis resulted in a separation between cyclic and pregnant animals (Fig 3C).

### RT-qPCR validation: miRNA regulation during early pregnancy

13 miRNAs (bta-miR-25, bta-miR-93, bta-miR-106b, bta-miR-125b, bta-miR-193a-5p, bta-miR-200b, bta-miR-200c, bta-miR-221, bta-miR-2898, bta-let-7i (MC); bta-miR-20a, bta-miR-29b, bta-miR-92 (SM)) which were found to be regulated during early pregnancy using NGS resutling, in case of MC miRNAs, in separation of cyclic and pregnant animals in PCA, were validated with RT-qPCR. The normalized Cq-values in qPCR correlated with the normalized NGS reads. Using the total expression level of average values from all samples, the significant correlation value in MC is -0.706 and -0.876 for SM, respectively. The correlation was also calculated for the values from all individual samples for each miRNA. With the exception of bta-let-7i in MC and bta-miR-20a in SM, a significant correlation was confirmed for all miRNAs including the housekeeping miRNAs.

Despite the overall corrrelation between NGS and qPCR samples, the regulation during early pregnancy could not be verified using qPCR. While the trends of regulation are clearly visible in technical validation, significances shown in NGS could only be confirmed for four miRNAs, which were either significantly regulated between pregnancy and oestrous cycle on day 4 (bta-miR-29b) or from day 4 to 18 between cycle and pregnancy (bta-miR-221, bta-miR-125b and bta-miR-200b). In biological verification the trend of the upregulated miRNAs (bta-miR-25, -miR-106b, miR-93, -let 7i) was confirmed while downregulated miRNAs did not exhibit the same trend (bta-miR-2898, bta-miR-193-5p, bta-miR-125b, bta-miR-200b and bta-miR-200c) (Table 4).

**Table 4:** Foldchanges/ratios of regulated miRNA during oestrous cycle (cy) and pregnancy (p) measured in RT-qPCR.

miRNA	Fold-change (RT-qPCR)									
	Technical validation					Biological validation				
	Day			Day		Day			Day	
	4p/4cy	12p/12cy	18p/18cy	18cy/4cy	18p/4p	4p/4cy	12p/12cy	18p/18cy	18cy/4cy	18p/4p
**Regulation in Milk Cells**										
bta-miR-25	1.15	2.36	1.66	-1.08	1.33	-1.20	1.02	1.27	-1.18	1.17
bta-miR-93	1.15	***1*.*80***	2.83	-1.16	1.90	1.20	1.11	1.72	1.02	1.16
bta-miR-106b	1.33	1.23	1.87	-1.05	1.29	-1.14	-1.52	1.16	-1.20	1.02
bta-mir-221	1.08	2.21	1.87	-1.07 **(a)**	1.59 **(a)**	1.17	-1.18	1.20	1.11	1.01
bta-let-7i	1.89	1.31	1.64	1.24	1.10	-1.25	-1.47	1.04	-1.69	-1.41
bta-miR-2898	3.71	2.08	1.00	2.18	-1.53	-1.37	-1.13	1.42	-1.31	1.85
bta-miR-193a-5p	3.90	2.66	-1.16	2.08	-1.83	-1.33	1.39	2.02	-1.32	1.44
bta-miR-125b	**3.41**	1.51	-1.37	2.40 **(a)**	-1.65 **(a)**	-1.41	-1.05	1.27	-1.06	1.30
bta-miR-200b	5.51	3.87	-1.24	3.11 **(a)**	-1.61 **(a)**	-1.52	1.17	**1.54**	-1.75	1.17
bta-miR-200c	4.79	3.70	-1.28	2.69	-1.77	-1.49	1.42	1.99	-1.79	1.38
**Regulation in Skim Milk**										
bta-mir-92a	-1.12	-1.40	1.08	-1.31	-1.08					
bta-mir-20a	1.08	1.11	1.04	1.10	1.01					
bta-mir-29b	**2.30**	**1.45**	1.39	1.63	1.12					

RT-qPCR fold-change/ratios was assessed with 2^-ddct^ from n = 6 animals for technical validation in skim milk and milk cells and n = 4 or n = 2 animals for biological validation in milk cells on days 4 and 18 or day 12, respectively. p-values were calculated using paired t-test on dct values. Significant values (p<0.05) are indicated in bold, highly significant values (p<0.01) in bold italic. Significant differences between corresponding groups are indicated with letter (a) in bold.

## Discussion

During the period of early pregnancy, fundamental changes are regulated in the maternal reproductive organs in order to protect and supply the embryo in an optimal way. Thereby, the immune system plays a pivotal role in allowing the semi-allograft embryo to survive. The aim of this study was to detect these changes in the form of miRNA regulation in two differing milk fractions for subsequent use as pregnancy detection biomarker.

In our study we found several potential pregnancy biomarkers, as discussed below. Each of these miRNAs was regulated either in SM or MC, respectively, but only two in both milk fractions. Thus, the miRNA profiles of the milk fractions were analysed to better understand the different mechanisms of miRNA entry into the milk. The composition of the milk miRNA profile was initially described in 2010 by Chen et al., but to our knowledge, a separate analysis of bovine MC and SM fraction is yet to be reported [40]. As both sample types in our study were isolated from bovine whole milk samples, we found a high consensus between our miRNA profiles and the reported bovine milk and mammary gland specific miRNAs [21, 40]. Similar to the findings of Alsaweed et al. 2015, in human breast milk miR-148a and miR-30a were highly abundant in both milk fractions, and were higher expressed in SM than in MC [43]. The miRNA profile was well preserved over all 36 MC and 35 SM samples, respectively. Therefore, it can be assumed that RNA degradation did not have major impact on the miRNA profile even though mRNA degradation products in the milk samples were likely, considering low RIN values and high numbers of sequencing products smaller 16 nt. Although the miRNA profile was similar between MC and SM, both milk fractions were distinct. On the one hand, MC were richer in miRNA species than SM, which only held 67% of the miRNAs compared to MC. On the other hand, MC and SM profile were also distinguishable due to immune cell related miRNAs which are significantly higher expressed in MC than in SM. Nevertheless, most of the reads in the MC belong to milk specific miRNAs, while the immune cell related miRNAs only make up a small fraction of the total miRNA reads. This is remarkable because somatic milk cells largely consist of immune cells entering the udder from the blood across the basal membrane in a process called diapedesis. Mammary epithelial cells are usually only detected in very low levels in bovine milk [44]. Thus, we expected a higher blood cell derived influence. However, this is consistent with data of a recent study by Alsaweed et al. 2016 which shows that the miRNA profile of milk cells and lipids were similar but did not relate to the profile of peripheral blood mononuclear cells or plasma [45]. From these findings the authors suggest that the majority of milk miRNAs originate from the same source, namely mammary epithelial cells. The hypothesis of Alsaweed et al. is supported by our data [45]. As mammary epithelial cells are glandular cells who’s key role is the secretion of milk components it could well be that most miRNAs originate from these cells rather than from the high number of immune cells [46]. It is however also possible that the immune cells change their expression profile as a result of the striking change in morphology which occurs due to uptake of milk fat globules and caseins when entering the milk [46].

In 2010 Hata et al. demonstrated that exosomal milk miRNAs, which comprise the majority of SM reads, are mainly secreted by the mammary gland [23]. The packaging of microvesicles and exosomes in particular, is a highly regulated process [47]. As the released miRNA composition does not reflect the cell internal miRNA composition, this may explain why some of the miRNAs found in high levels both in MC and SM do not correlate amongst these two sample types deriving from identical whole milk samples. Even though the milk-derived influence predominates the miRNA profile in MC, we assume that the physiological state of the animal is better reflected in this milk fraction. On the one hand MC are richer in miRNAs species than SM in our study on bovine milk as well as in the human milk lipid fraction in a further study of Alsaweed et al. [48]. On the other hand not only about 2.5 times more miRNAs were found to be regulated than in SM, but theses miRNAs largely also showed a more distinct trend of regulation during the course of oestrous or pregnancy, respectively. Most likely the reason for this is the exposure of the cells to many signals, including pregnancy-related ones, during trafficking through the maternal system while the majority of exosomes in the SM are secreted by mammary gland cells in an accurately defined way to ensure nutrient quality for infants. This leads not only to less diversity than in MC samples, but probably also to less information about the physiological state of the mother. It might however also be possible that the lower total miRNA readcounts in SM may impair the sensitivity for expression change detection compared to the MC fraction.

We propose two possible mechanisms through which differentially expressed miRNAs related to pregnancy are present in milk: 1^st^ due to direct secretion of the miRNAs, most likely protected by microvesicles, entering from the uterine tract into the blood circulation and consequently into milk or 2^nd^ by induction of miRNA expression in blood cells or the udder itself due to stimulating hormones such as progesterone or direct or indirect effects of the bovine recognition signal interferon tau [49, 50].

In the search for biomarkers in tissues spatially remote from the reproductive organs, part of the pregnancy-induced expression changes of miRNAs can be restricted in their entry into the udder or masked by the high number of milk-derived miRNAs. Thus, the expression differences between oestrous cycle and pregnancy were rather unpronounced. Additionally, every animal showed a distinct expression profile which was, referring to Li et al. 2012, due to lactation and the physiological state of the animals [51]. We therefore did not detect major expression changes at single time points but rather trends in regulation over a period of time. Nevertheless, many potential biomarkers we found (MC: Bta-miR-25, -106b, -93, -221, -223 -193a-5p, -125a/b, -200c, -200b, bta-let-7i; SM: Bta-miR-92a, -20a, -29b/c) have been described to be involved in pregnancy in earlier studies [17, 18, 52–54].

Five miRNAs belonging to the miR 17~92 cluster and its paralog cluster 106b~25 attracted our particular interest. The evolutionarily-related clusters are encoded on the bovine chromosomes 12 and 25, respectively and encode miRNAs share the same seed sequences [55]. The miRNAs are connected to mis-regulation in cancer [56] as they are potentially involved in the regulation of pluripotency in stem cells [57] and angiogenesis [58, 59]. However, these processes are also crucial for reproductive success. Thus, it is not surprising that several studies found members of the clusters playing key roles in trophoblast differentiation [52], placental diseases [59–61] and embryonic development [4, 18, 62]. The cluster 106b~25 holds three miRNAs (bta-miR-106b, -93, -25). All three miRNAs showed an increase in expression during early pregnancy in MCs in the high throughput screening. Additionally in SM we found not only differential expression of miR-25 on day four of pregnancy but also of two (bta-miR-20a, -92a) of the six polycistronical miRNAs encoded on cluster 17~92. Interestingly, bta-miR-29b has been shown by Li et al. to have similar actions in human pregnancy as cluster 17~92 and is clustered with miR-29c that has shown the same expression pattern in our study as miR-29b [17, 63]. The accumulated discovery of regulation of these miRNAs in our sequencing study contributes to the hypothesis that their involvement in the uterine tract is detectable in milk. In contrary to the members of cluster 106b~25, the two polycistronic miRNAs bta-miR-20a and -92 did not follow the same expression trend. Guo et al., who also observed inconsistent expression levels in their studies, explained the phenomenon with the complex regulatory or degradation mechanisms that can lead to varying expression levels [55, 64, 65]. Curiously, several miRNAs in our study, including miRs-29b/c, miR-25 and miR-140 are significantly regulated on day four of pregnancy. At this time the fertilized oozyte is entering the uterus after its journey through the oviduct. To date no systemic recognition of pregnancy is known by the maternal environment at this state [26]. Whether our results are a clear indication of an early embryo-oviductal interaction remains to be substantiated.

The MC fraction contained several more regulated miRNAs during early pregnancy. miR-221 for example, is not only a miRNA known to be expressed by immune cells but has also been described to influence endometrial stroma cell decidualization, oocyte and follicle development [53, 66–68]. The miR-200 family, despite beeing highly expressed in milk, has been found to be differentially expressed in endometrial and oocyte development [60, 67, 69]. miR-193a-5p is involved in the endometrial and oocyte development [53, 70] and miR-223 and miR-125a have been found in extracellular uterine fluid vesicles [54]. The closely-related miRNA miR-125b, which is encoded in a cluster with miR-100 which is also down-regulated at day 18 of pregnancy, has been shown to be differentially expressed in the endometrium and placenta and may also be involved in the endometrial immune response [60, 67, 69, 71]. Additionaly, bta-miR-125b was found to be down-regulated in bovine plasma in a recent study by Ioannidis et al. between day 24 pregnant and non-pregnant heifers [72]. The same study both identified and biologically-validated bta-miR-26a as differentially up-regulated between day 24 pregnant and non-pregnant heifers [72]. This miRNA was slightly but significantly up-regulated in our study as well on day 18 pregnant vs. day 18 cyclic together with a member of the same family (bta-miR-26b) that also showed significant expression changes from day 4 to 18 between pregnancy and cyclic state in the MC fraction.

Interestingly, according to our sequencing data, all the afore-mentioned miRNAs in MCs and some in SM showed up-regulation during oestrus cycle from day 4 to day 18 but exhibited the contrary regulation during pregnancy and vice versa. This may indicate their impact in the important adaption to the new physiological state of the mother. Using day 18 to day 4 ratio in MC sequencing data of a set of ten miRNAs (bta-miR-25, bta-miR-93, bta-miR-106b, bta-miR-125b, bta-miR-193a-5p, bta-miR-200b, bta-miR-200c, bta-miR-221, bta-miR-2898, bta-let-7i) discrimination between pregnant and cyclic animals was achieved in a PCA. This would identify their potential as a pregnancy biomarker set. However, these findings were neither satisfactorily technically nor biologically validated on an independent platform by using RT-qPCR. Other research groups have previously reported similar difficulties with technical validation [12, 20]. One must consider that qPCR and NGS data are not only of a different type (NGS is an absolute and qPCR a relative method of quantification) but the data of both methods are normalized by different procedures. While the readcounts of NGS are normalized based on the median-of-ratios method [35], qPCR normalization is performed on the base of stable expressed reference genes [38]. Especially in the case of small expression changes between sample groups, as in our data, the same results may not always be obtained on both quantification platforms. The biological validation showed remarkable differences to the regulations found in NGS sequencing for some miRNAs. Only a larger sample size could determine whether the four animals used for biological validation were random outliers by chance.

## Conclusion

In this study we compared the miRNAs of MC and SM samples for their use as biomarkers in early pregnancy detection of cattle. The miRNA profiles were surprisingly similar between MC and SM. The miRNA profile of MC from a milk liquid biopsy more accurately reflected the physiological state of the animal for use as a biomarker than that of SM. This was probably due to their closer connection to the maternal blood circulation, resulting in more regulated miRNAs in this sample group. Additionally, the MC data of the high-throughput sequencing could be used to differentiate between cyclic and pregnant cows. Currently, we cannot recommend the analysed biomarkers from this study for diagnostic use. On the one hand the RT-qPCR validation was not satisfactory while on the other hand a single time point analysis in a routine test would be preferable over a subsequent sample drawing as indicated by our data.

## Supporting information

S1 Table miRNAs > 50 reads. All miRNAs with more than 50 reads found in MC and/or SM are listed. Mean readcounts of all 36 MC or 35 SM samples and the corresponding ratio to all miRNA reads are listed. The variation of the ratio among the samples is shown as SEM.
(XLSX)

S2 Table Significantly expressed miRNAs in milk cells between pregnancy and cycle. miRNAs with significant fold-changes/ratios either between pregnancy and cycle on days 4, 12 or 18 (p-value derived from Deseq2) and/or between the expression change from day 4 to 18 change of cycle and pregnancy (p-value derived from paired t-test between fold-changes/ratios of pregnant and cyclic group).
(XLSX)

S3 Table Significantly regulated miRNAs in milk cells during cycle and/or pregnancy. miRNAs with significant fold-changes/ratios in the process of cycle and/or pregnancy from day 4 to 18. p-value are derived from Deseq2.
(XLSX)

S4 Table Significant different miRNA expression levels in skim milk between pregnancy and cycle. miRNAs with significant fold-changes/ratios either between pregnancy and cycle on days 4, 12 or 18 (p-value derived from Deseq2) and/or between the expression change from day 4 to 18 of cycle and pregnancy (p-value derived from paired t-test between fold-changes/ratios of pregnant and cyclic group).
(XLSX)

S5 Table Significantly regulated miRNAs in skim milk during cycle and/or pregnancy. miRNAs with significant fold-changes/ratio in the process of cycle and/or pregnancy from day 4 to 18. p-value are derived from Deseq2.
(XLSX)

S6 Table Progesterone concentrations. Progesterone Concentrations [ng/ml] measured in milk according to Prakash et al. 1987 [28]. Concentrations were used for cycle and pregnancy control.
(XLSX)
